# Supplementary material for: Fractionation of Enriched Phosphopeptides Using pH/Acetonitrile-Gradient-Reversed-Phase Microcolumn Separation in Combination with LC–MS/MS Analysis
Source: Int J Mol Sci. 2020 Jun 1;21(11):3971. doi: 10.3390/ijms21113971 (PMC7312998; doi:10.3390/ijms21113971)
Supplement: Supplementary file 1 [file ijms-21-03971-s001.pdf]

International Journal of *Molecular Sciences*

MDPI

Figure S1. Description of our in-house apparatus.

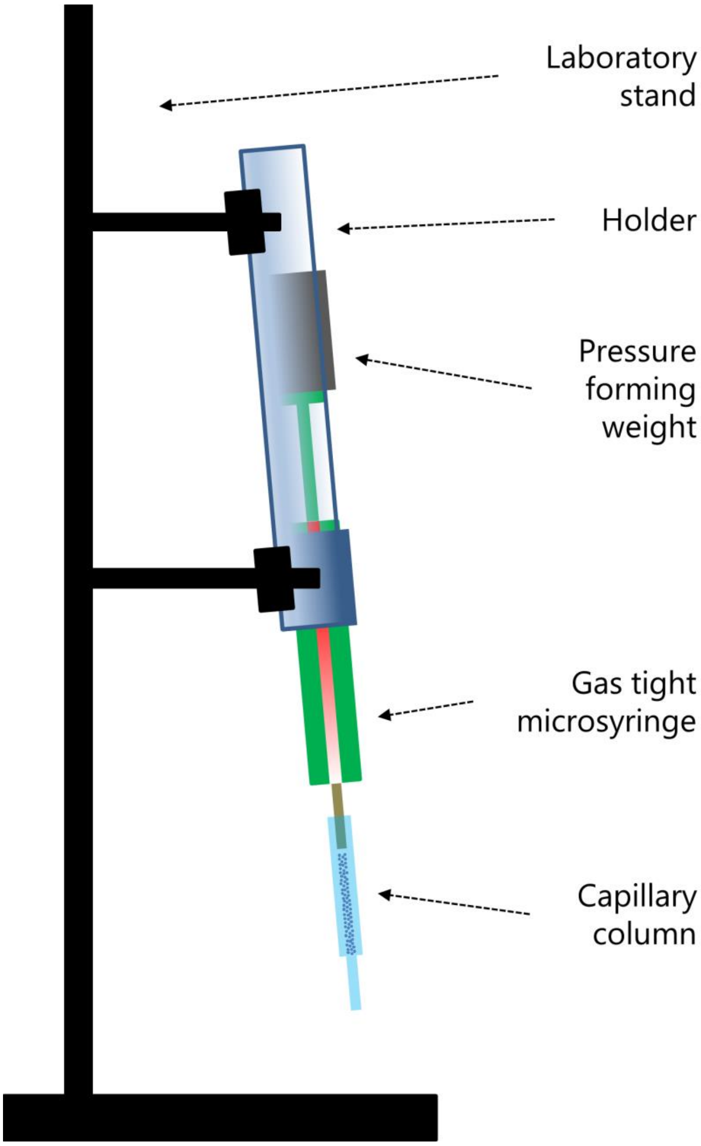

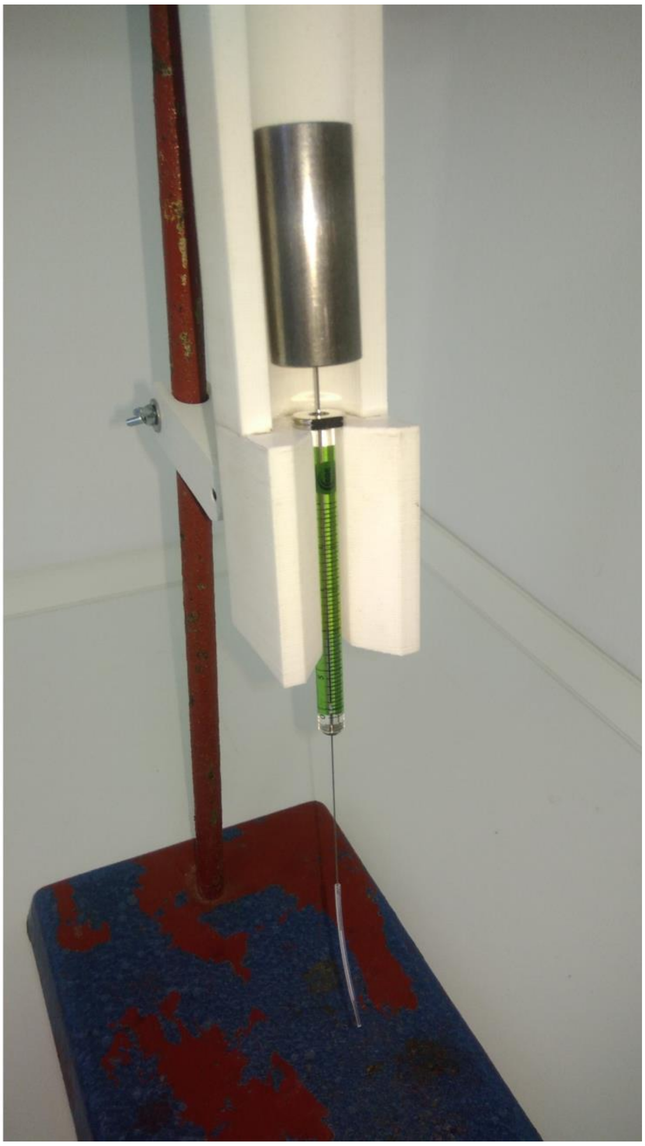

Overall description of our in-house made microcolumn apparatus. The whole apparatus consists of: i) a laboratory stand, ii) a holder that holds a microsyringe and a pressure forming weight, iii) metal pressure forming weight that creates constant pressure and thus ensures continuous elution, iv) gas tight microsyringe with a mobile phase, and v) capillary microcolumn composed of the fluorinated ethylene propylene tubing (1/16″ × 0.25 mm ID) filled with reversed-phase C18 microparticles (Kinetex EVO C18 2.6 µm core shell particles).

Description of the capillary microcolumn. The outer tubing of our capillary microcolumn is made of the fluorinated ethylene propylene tubing (1/16″ × 0.25 mm ID). The total length of the tubing is about 50 mm. One of the ends is tapered and forms a narrower part. The wider part is then about 30–35 mm long and the narrower about 15–20 mm long. A glass filter (Whatman® Grade GF/A: 1.6 µm) sits at the bottom of the narrower part. The stationary phase C18 microparticles (Kinetex EVO C18 2.6 µm core shell particles) suspended in acetonitrile is loaded into the column using a 10µL pipette and the microparticles are pushed down using the airflow pressure created using a Combitip (Eppendorf). If needed additional volume of stationary phase is added to obtain the appropriate column length of 25 mm.

Decription of the elution process. Steel weights weighing 75 and 150 g are used to create a constant pressure. Using the steel weight of 75 g, the elution time is about 20 minutes and using the weight of 150 g, the elution time is about 10 minutes.

Figure S2. Distribution of phosphopeptides into the fractions by high-pH-RP fractionation

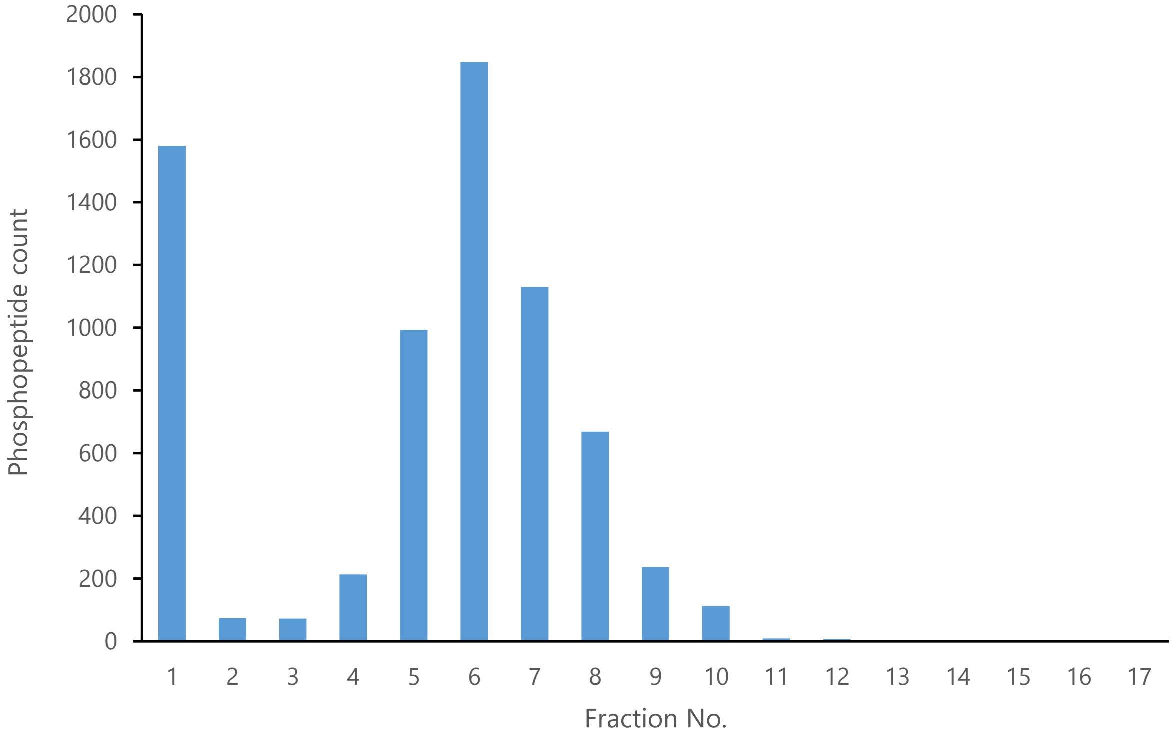

Sample description

The phosphopeptide mixture was fractionated using the high-pH-fractionation on the reversed phase (RP) as described in the Materials and Methods section, eluting into 17 fractions:

- fraction 1: flow-through from sample loading step
- fraction 2 to fraction 17: high pH fractions from reversed phase separation

LC-UV analysis of all the fractions is shown in the manuscript as Figure 2.

Summary information from the high-pH-RP fractionation

Summary statistics for the high-pH-RP fractionated sample of enriched phosphopeptides

- number of identified
  - peptides in all fractions: 7695
  - phosphopeptides in all fractions: **5941**
  - phosphopeptides in the fraction 1: **1580**
  - non-phosphorylated peptides in the fraction 1: 363
  - phosphopeptides in the fractions 2 to 17: 4446
  - non-phosphorylated peptides in the fractions 2 to 17: 1400
  - phosphopeptides in the fraction 1 identified also in some of the fraction 2 to 17: 85
  - phosphopeptides exclusively identified in the fraction 1: **1495**
- about 26.6% (= 1580/5941) of phosphopeptides eluted in the fraction 1 (flow-through)
- about **25.2%** (= 1495/5941) of phosphopeptides eluted in the fraction 1 exclusively and were not identified elsewhere
- sum of peak areas from LC-MS data assigned to peptide sequences in the fraction 1 for
  - phosphopeptides: 117,693,666,111
  - non-phosphorylated peptides: 39,877,434,562
- ratio of TIC area for phosphopeptides and non-phosphorylated peptides in the fraction 1: **2.95**

Figure S3.

Nano LC–UV chromatograms of individual fractions originating from the pH/acetonitrile-gradient-reversed-phase fractionation of the TiO_2-enriched phosphopeptide sample in three replicates. The corresponding fractions compared across all three replicates show good reproducibility of the fractionation method.

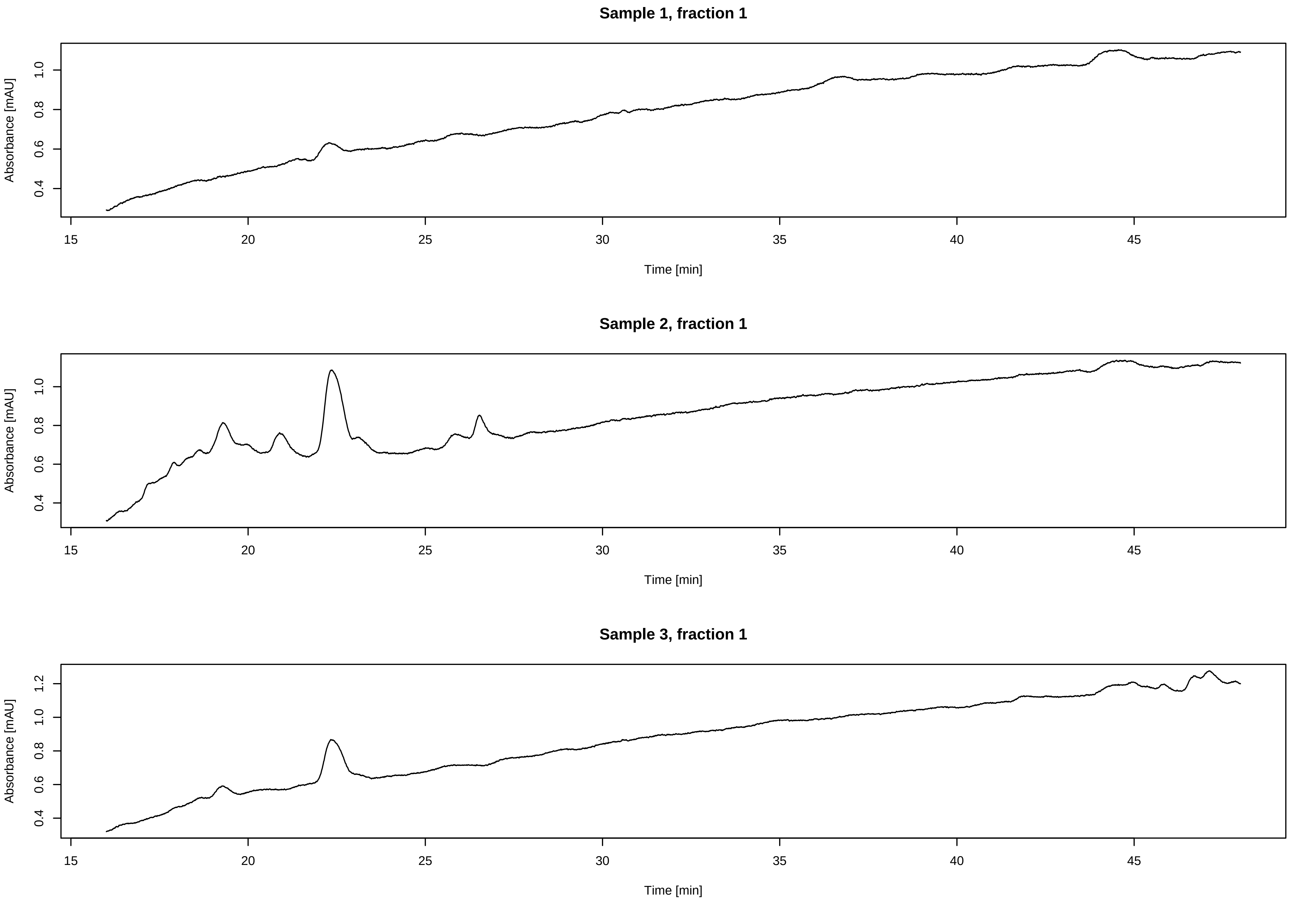
Sample 1, fraction 1
Sample 2, fraction 1
Sample 3, fraction 1
Absorbance [mAU]
Time [min]
0.4
0.6
0.8
1.0
1.2
15
20
25
30
35
40
45

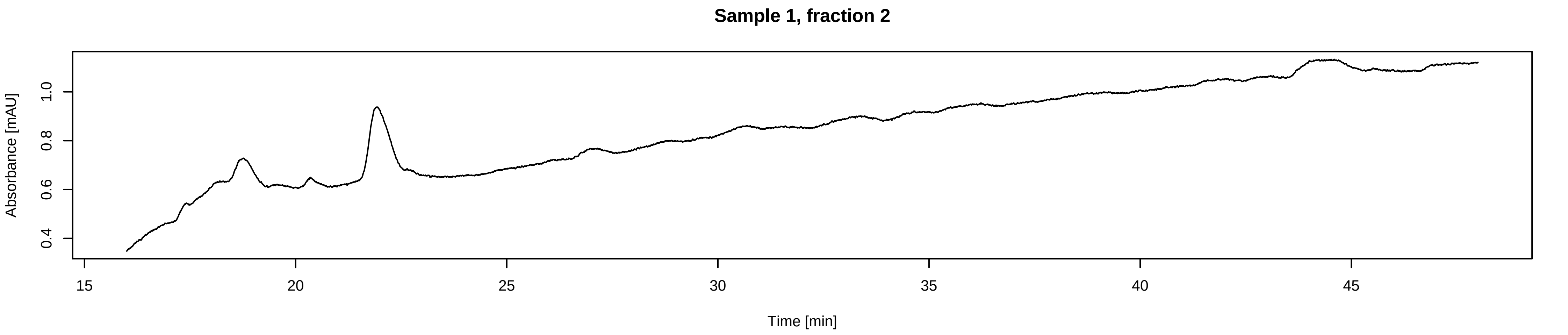
Sample 1, fraction 2
Absorbance [mAU]
0.4
0.6
0.8
1.0
15
20
25
30
35
40
45
Time [min]

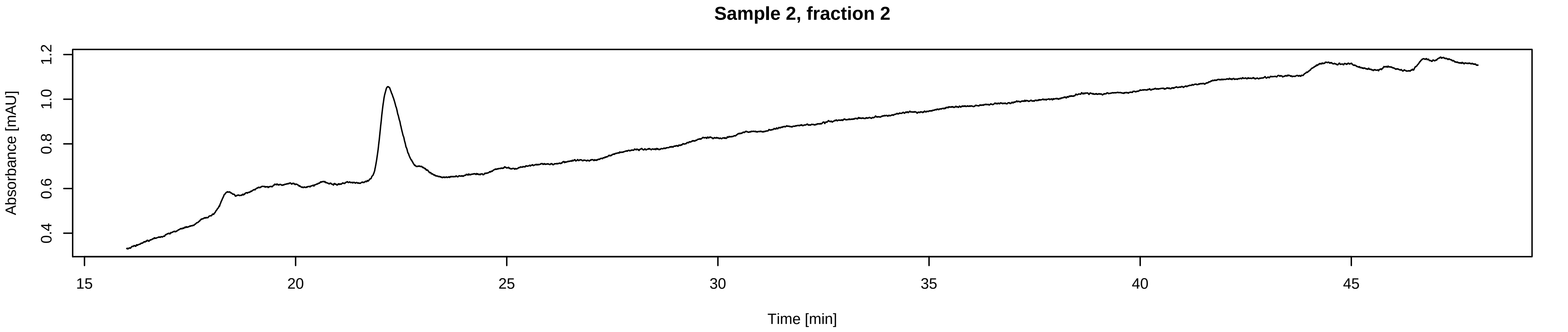
Sample 2, fraction 2
Absorbance [mAU]
0.4
0.6
0.8
1.0
1.2
15
20
25
30
35
40
45
Time [min]

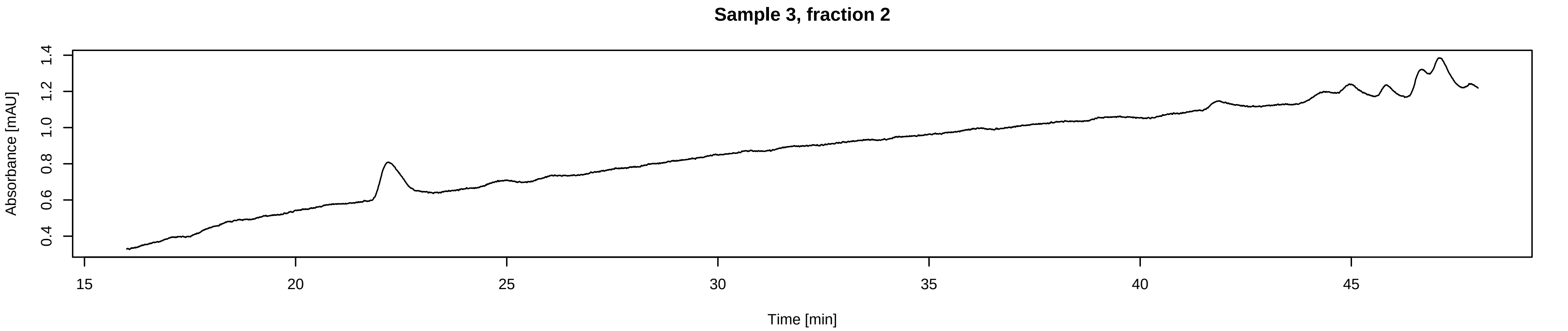
Sample 3, fraction 2
Absorbance [mAU]
0.4
0.6
0.8
1.0
1.2
1.4
15
20
25
30
35
40
45
Time [min]

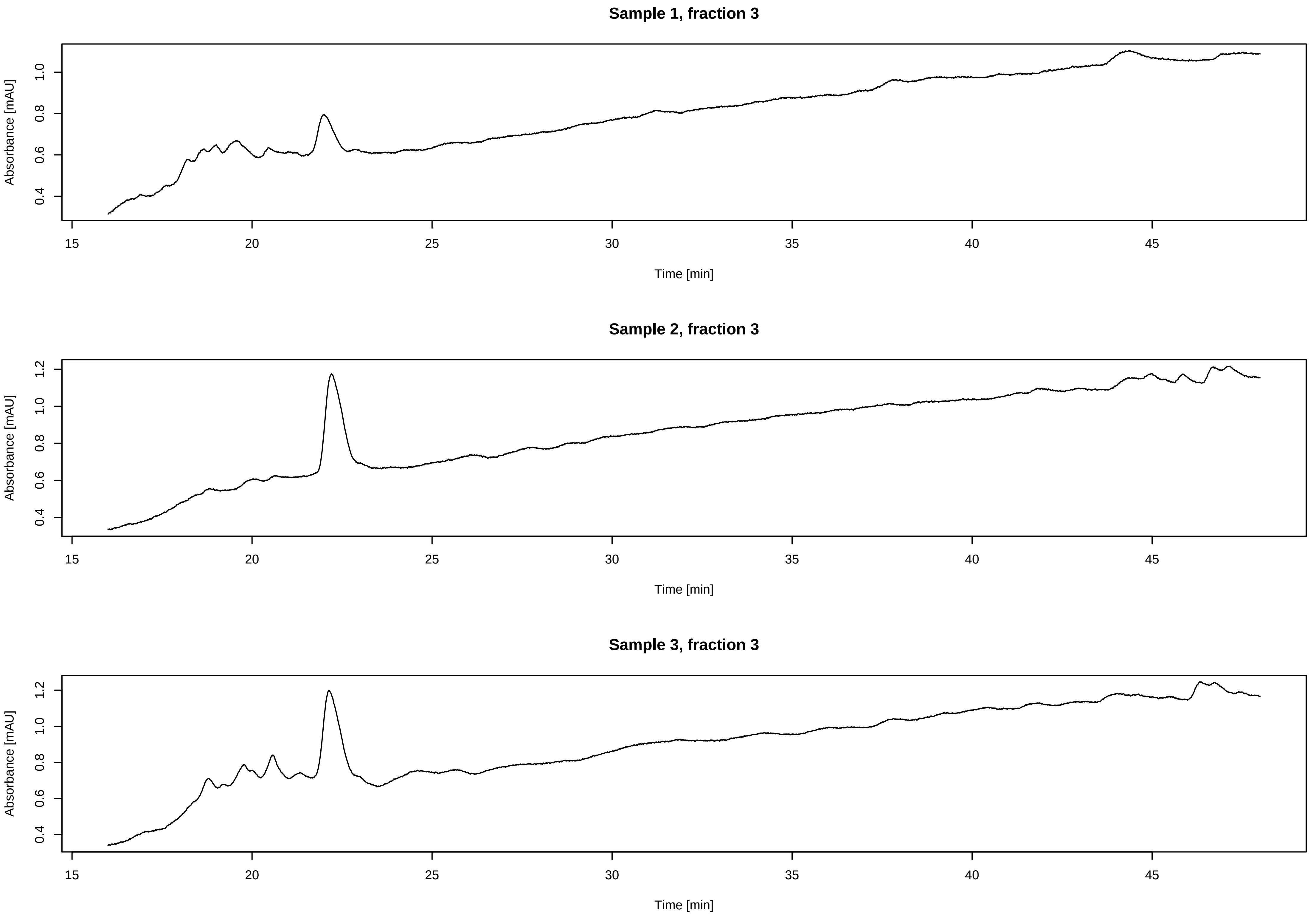

Sample 1, fraction 3
Absorbance [mAU]
0.4
0.6
0.8
1.0
15
20
25
30
35
40
45
Time [min]
Sample 2, fraction 3
Absorbance [mAU]
0.4
0.6
0.8
1.0
1.2
15
20
25
30
35
40
45
Time [min]
Sample 3, fraction 3
Absorbance [mAU]
0.4
0.6
0.8
1.0
1.2
15
20
25
30
35
40
45
Time [min]

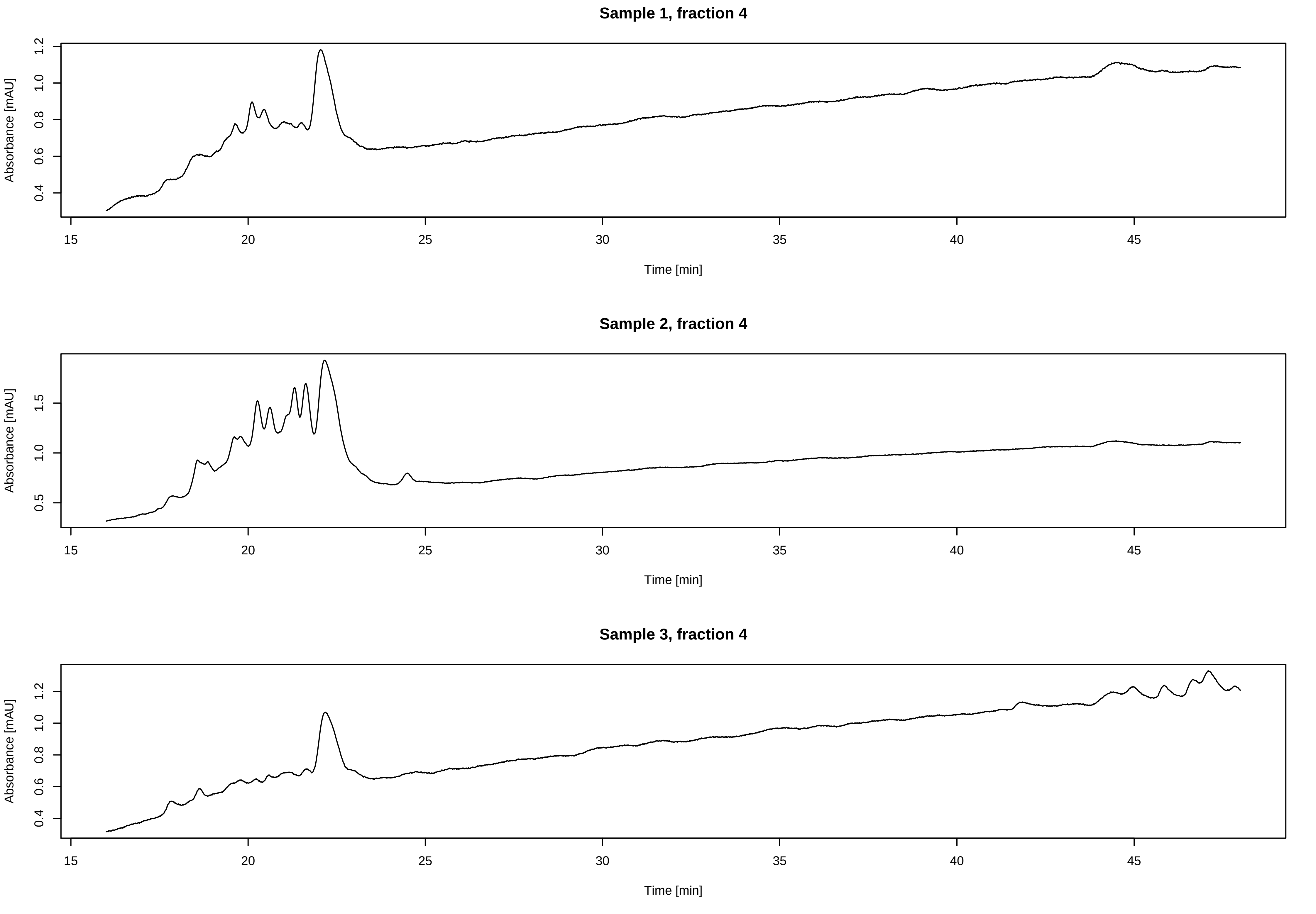

Sample 1, fraction 4
Absorbance [mAU]
Time [min]
Sample 2, fraction 4
Absorbance [mAU]
Time [min]
Sample 3, fraction 4
Absorbance [mAU]
Time [min]

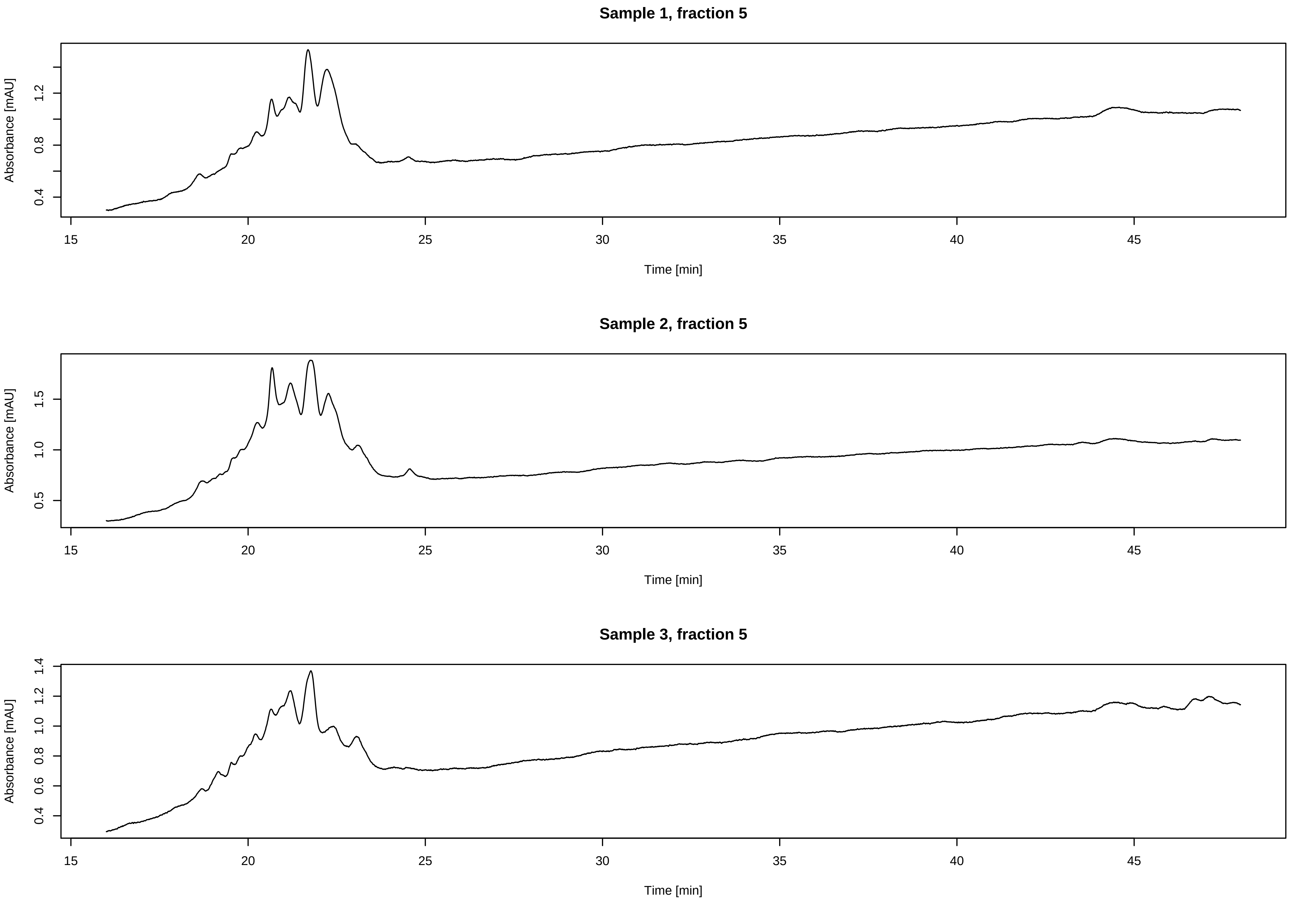
Sample 1, fraction 5
Absorbance [mAU]
0.4
0.8
1.2
15
20
25
30
35
40
45
Time [min]
Sample 2, fraction 5
Absorbance [mAU]
0.5
1.0
1.5
15
20
25
30
35
40
45
Time [min]
Sample 3, fraction 5
Absorbance [mAU]
0.4
0.6
0.8
1.0
1.2
1.4
15
20
25
30
35
40
45
Time [min]

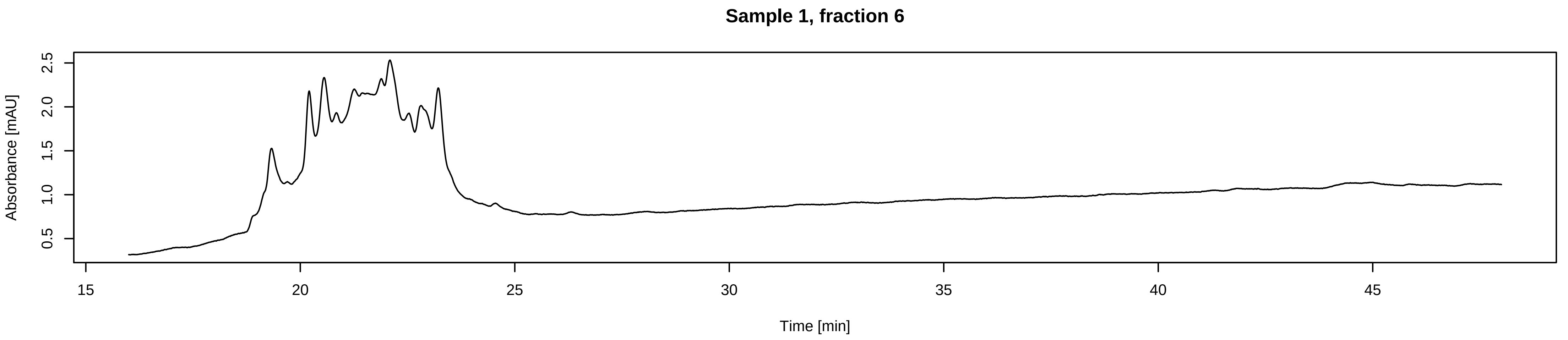
Sample 1, fraction 6
Absorbance [mAU]
0.5
1.0
1.5
2.0
2.5
15
20
25
30
35
40
45
Time [min]

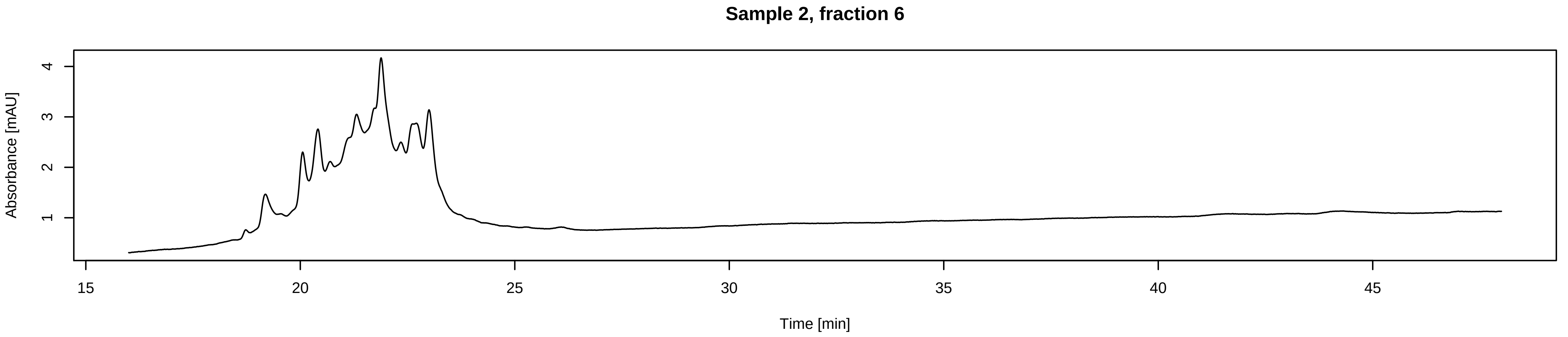
Sample 2, fraction 6
Absorbance [mAU]
1
2
3
4
15
20
25
30
35
40
45
Time [min]

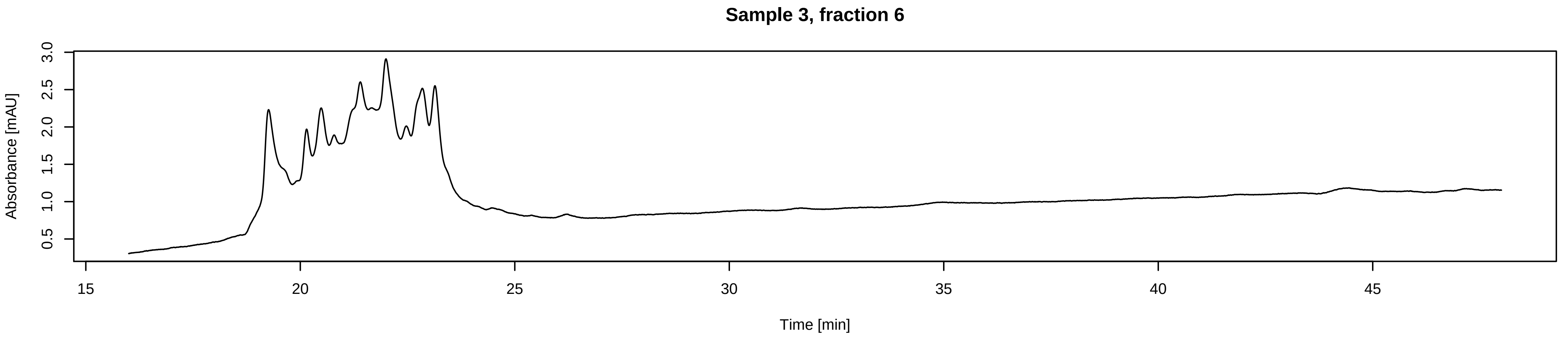
Sample 3, fraction 6
Absorbance [mAU]
0.5
1.0
1.5
2.0
2.5
3.0
15
20
25
30
35
40
45
Time [min]

Sample 1, fraction 7

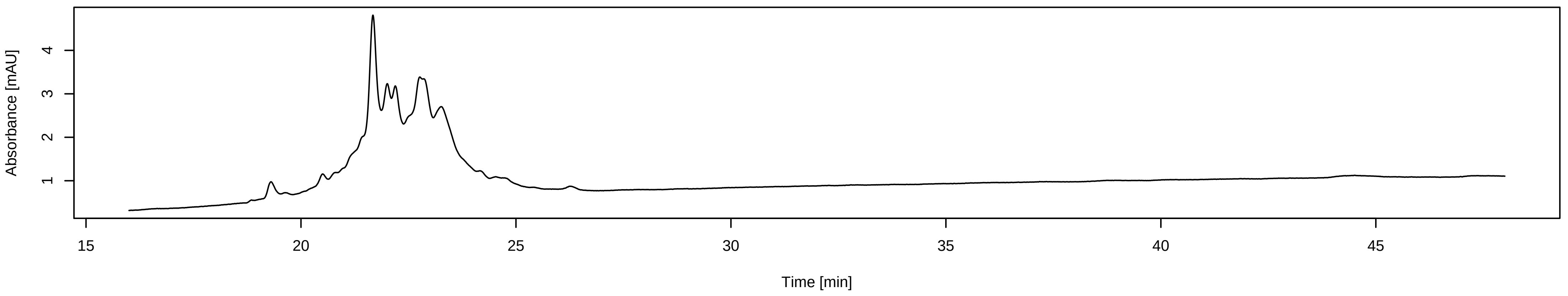

Sample 2, fraction 7

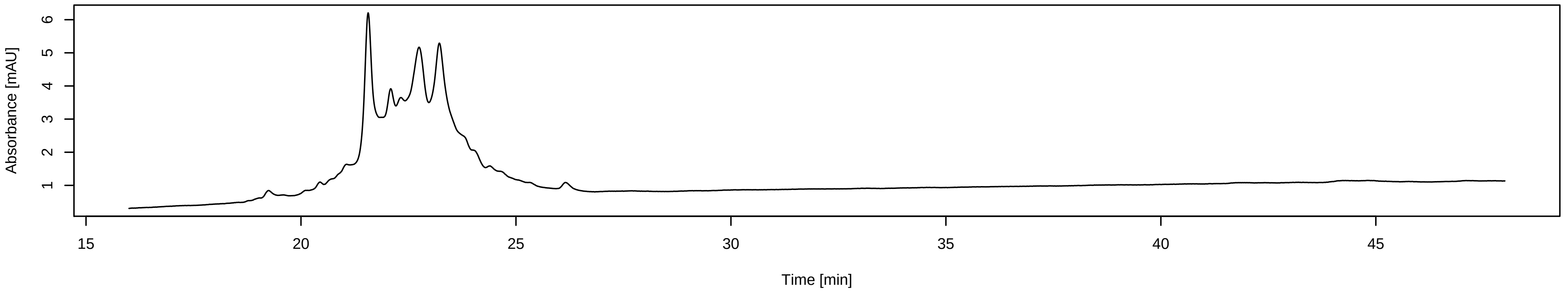

Sample 3, fraction 7

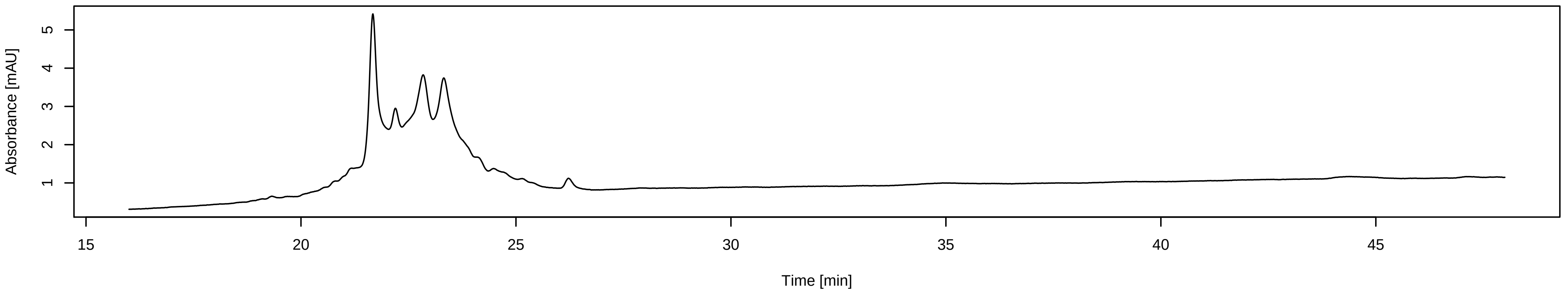

Sample 1, fraction 8

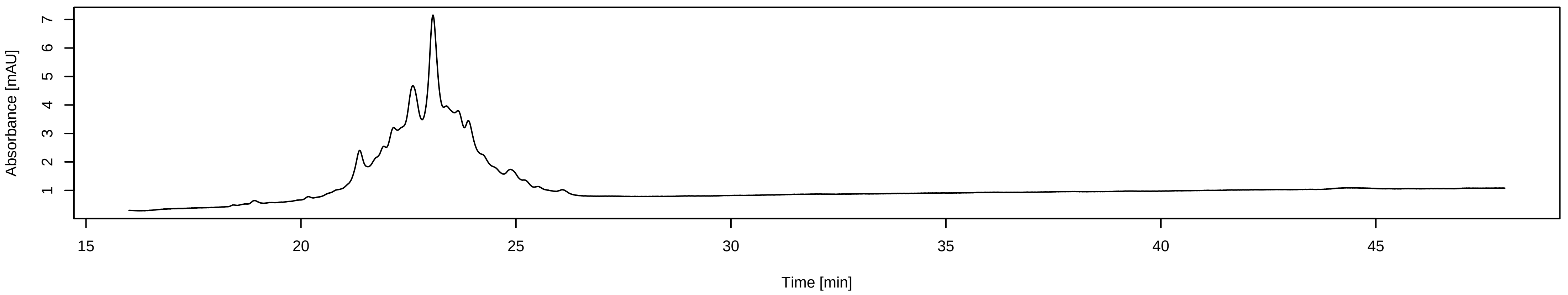

Sample 2, fraction 8

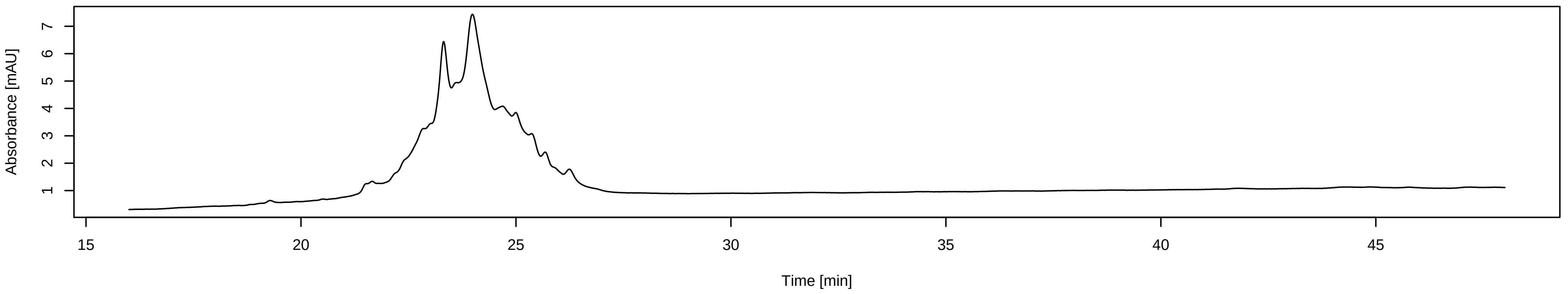

Sample 3, fraction 8

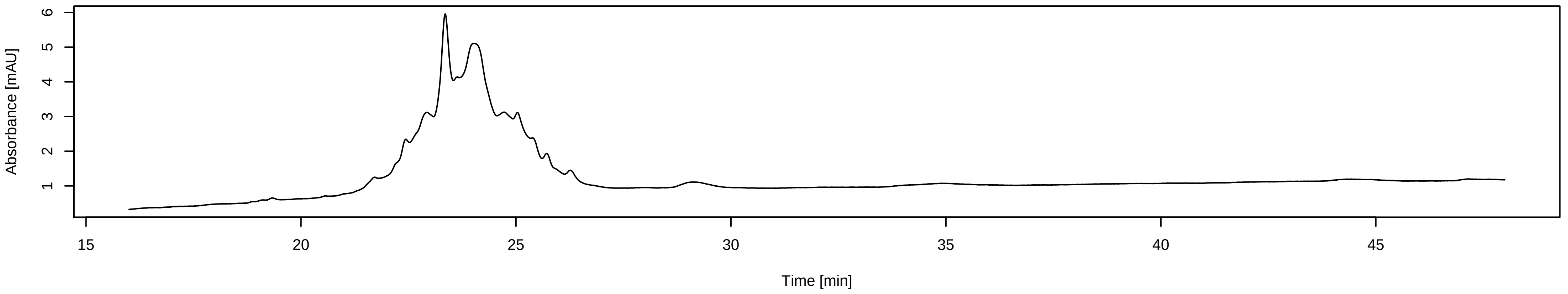

Sample 1, fraction 9

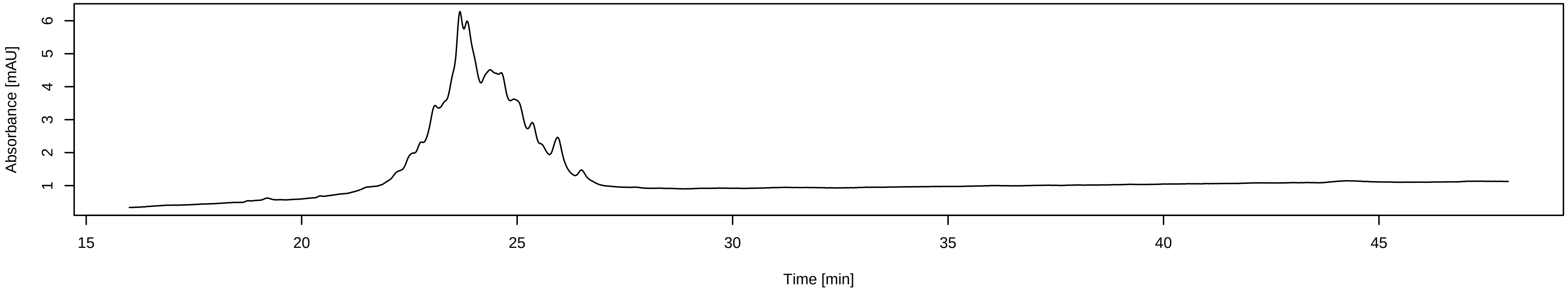

Sample 2, fraction 9

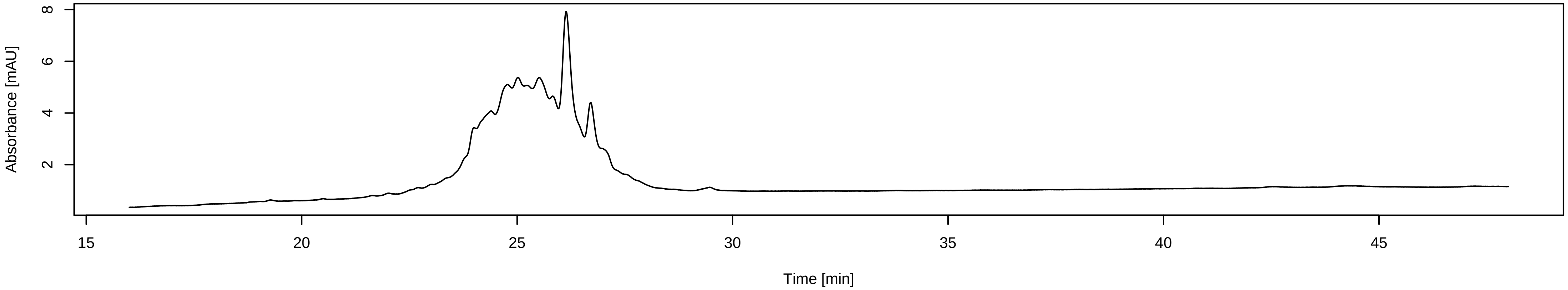

Sample 3, fraction 9

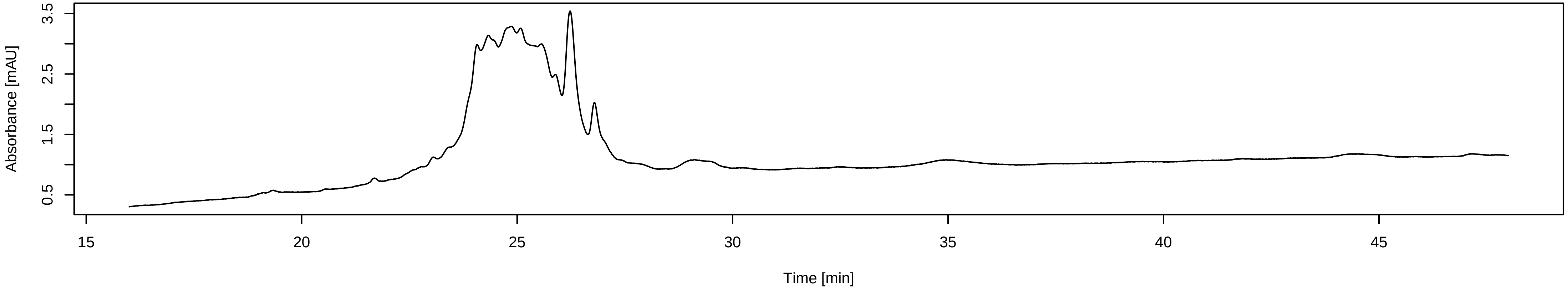

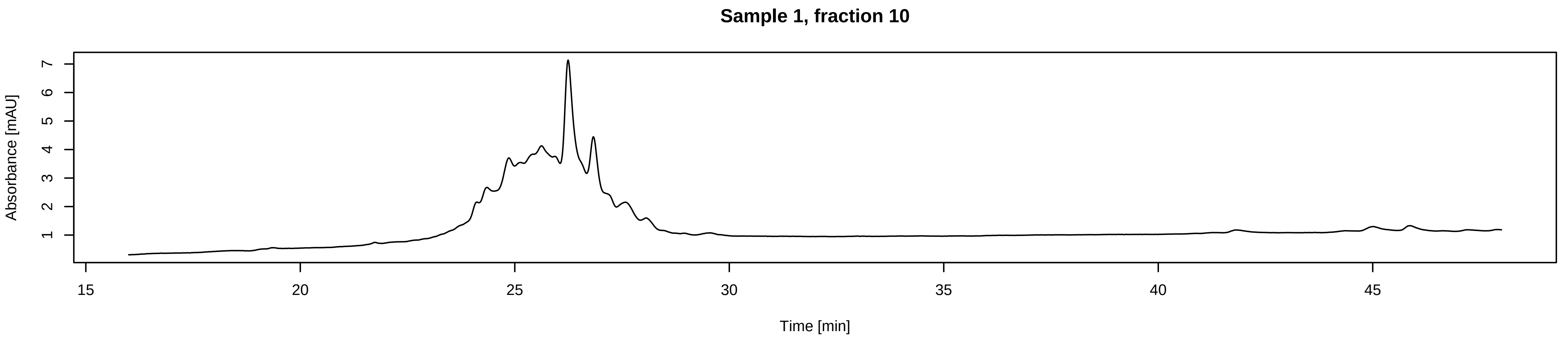
Sample 1, fraction 10
Absorbance [mAU]
Time [min]
15
20
25
30
35
40
45
1
2
3
4
5
6
7

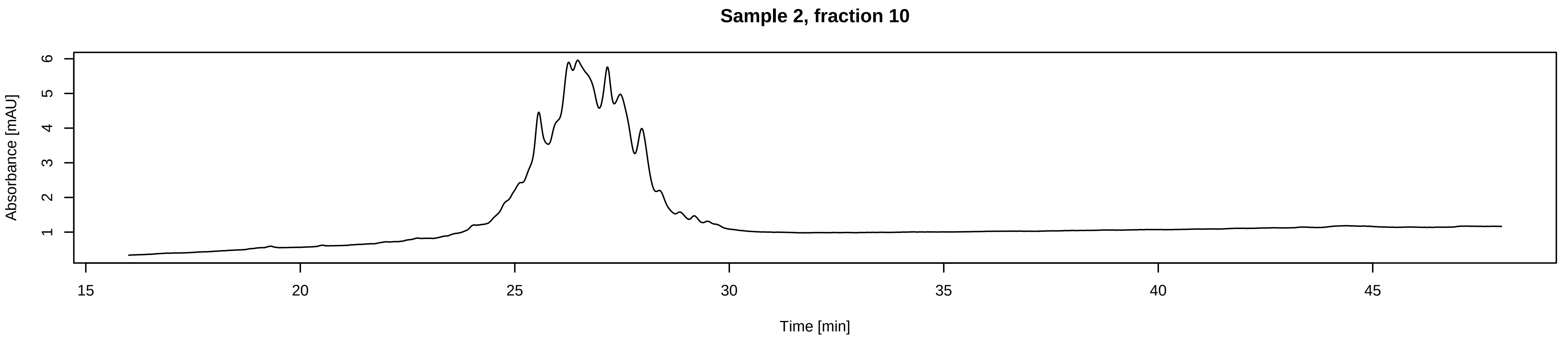
Sample 2, fraction 10
Absorbance [mAU]
Time [min]
15
20
25
30
35
40
45
1
2
3
4
5
6

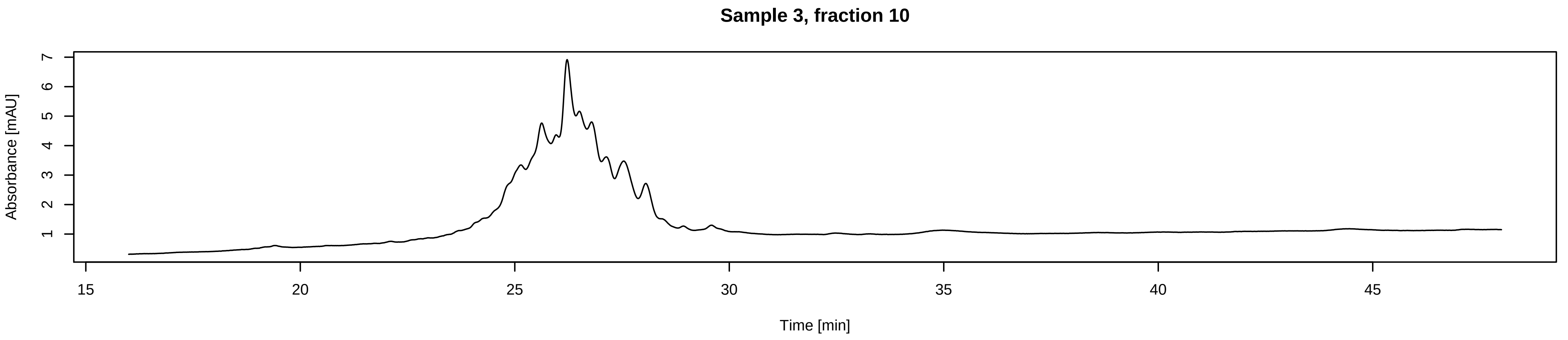
Sample 3, fraction 10
Absorbance [mAU]
Time [min]
15
20
25
30
35
40
45
1
2
3
4
5
6
7

Sample 1, fraction 11

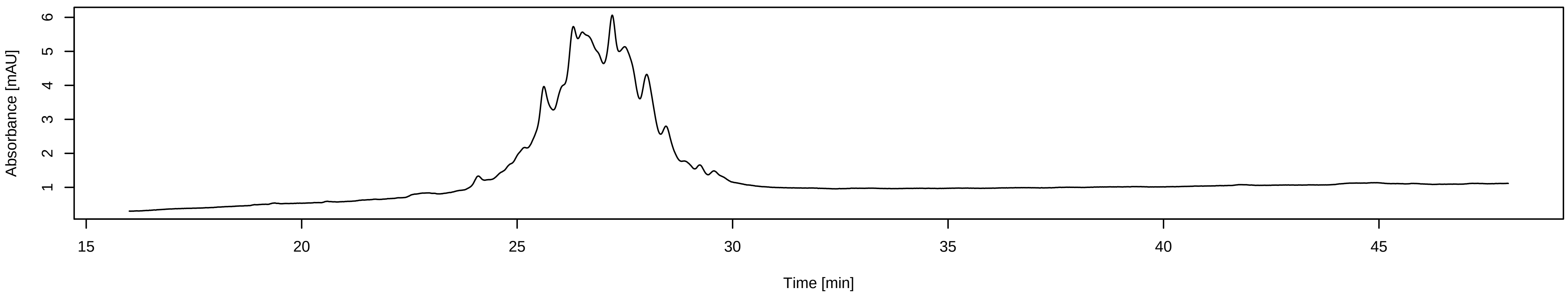

Sample 2, fraction 11

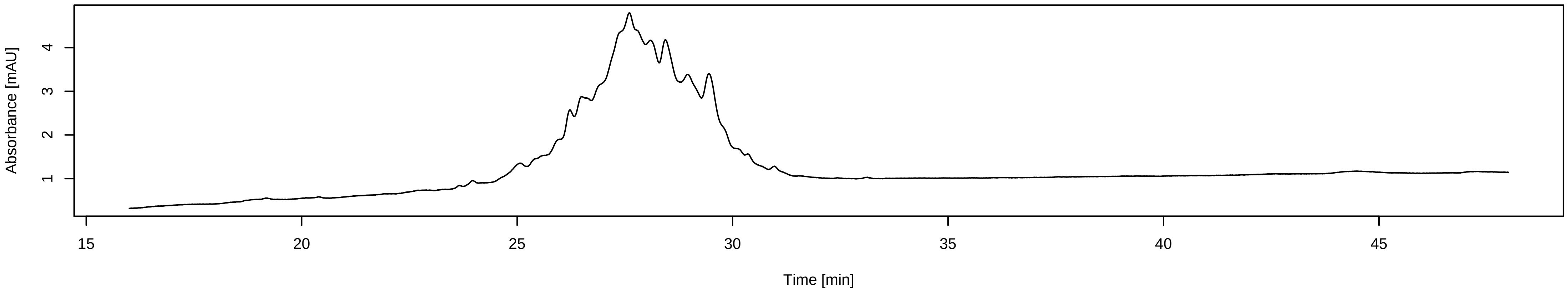

Sample 3, fraction 11

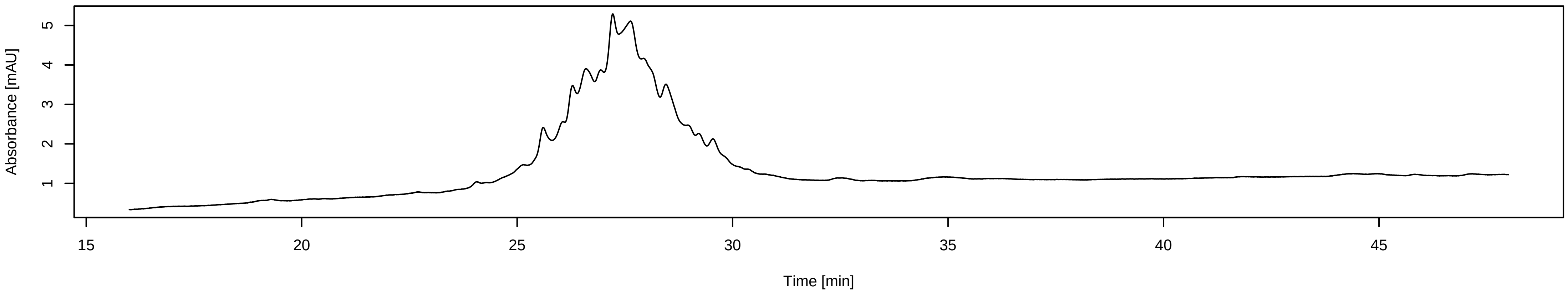

Sample 1, fraction 12

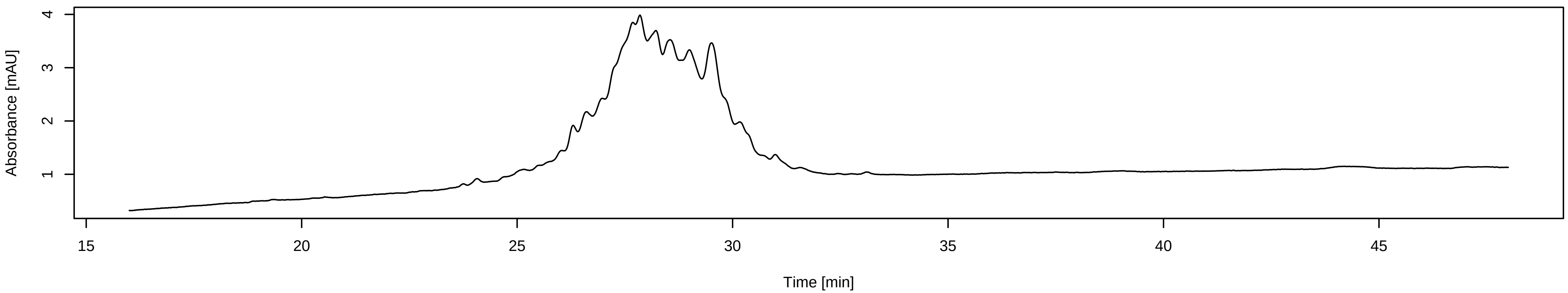

Sample 2, fraction 12

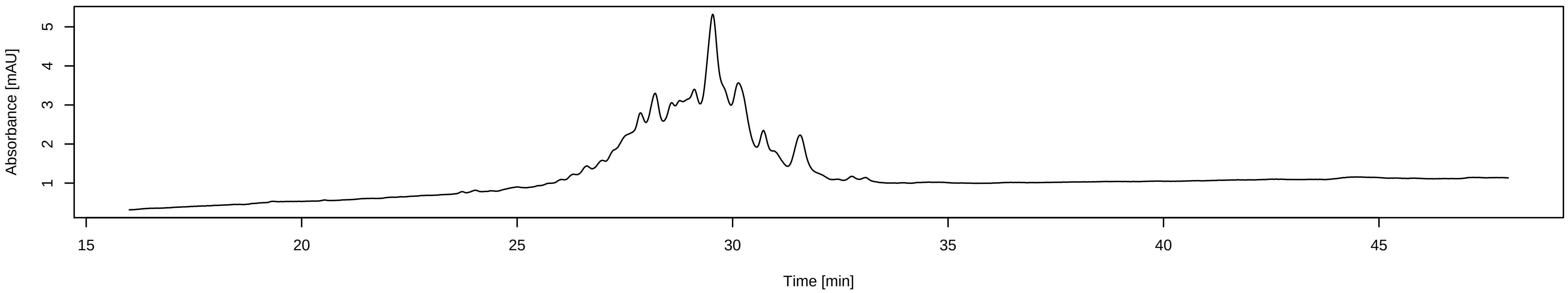

Sample 3, fraction 12

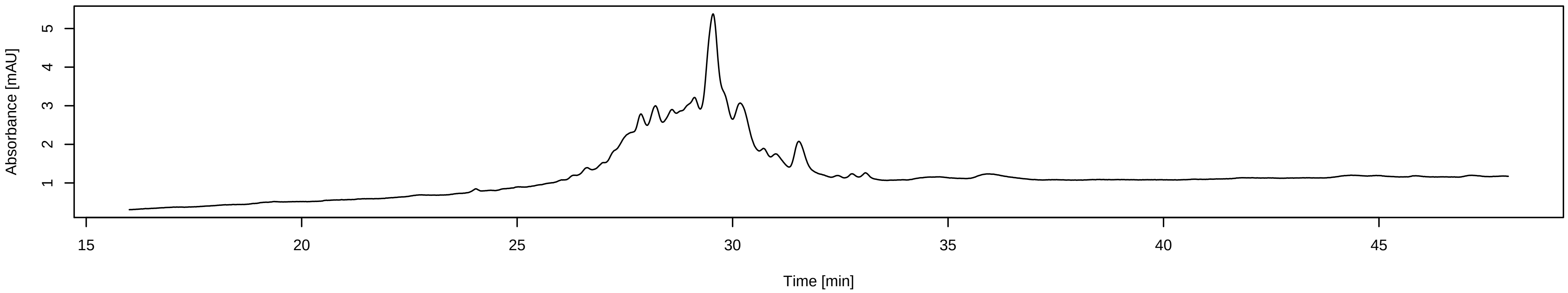

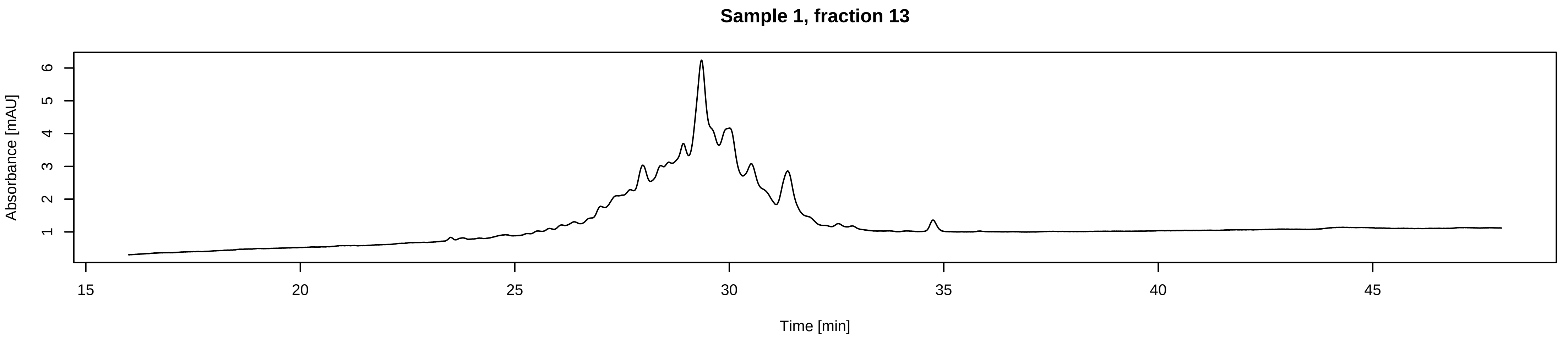
Sample 1, fraction 13
Absorbance [mAU]
1
2
3
4
5
6
15
20
25
30
35
40
45
Time [min]

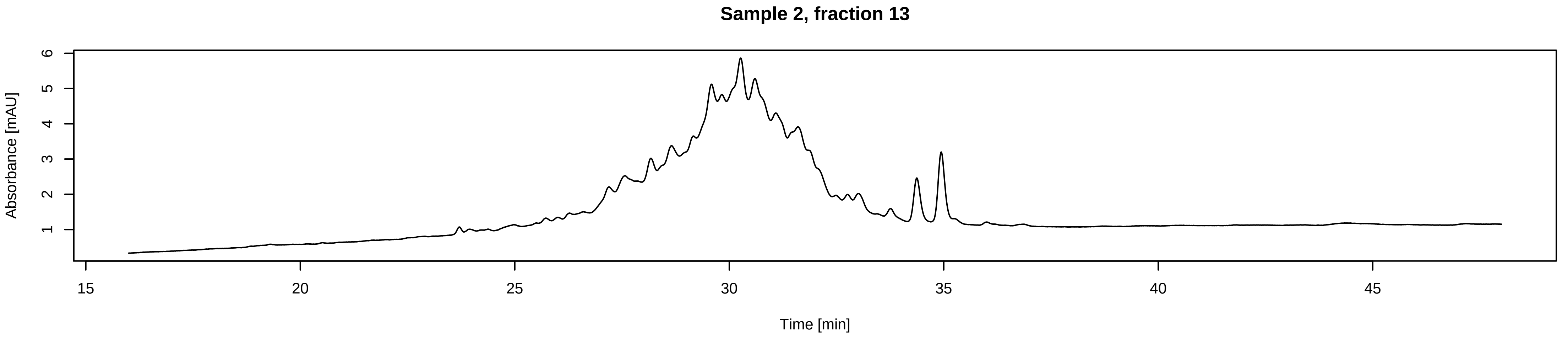
Sample 2, fraction 13
Absorbance [mAU]
1
2
3
4
5
6
15
20
25
30
35
40
45
Time [min]

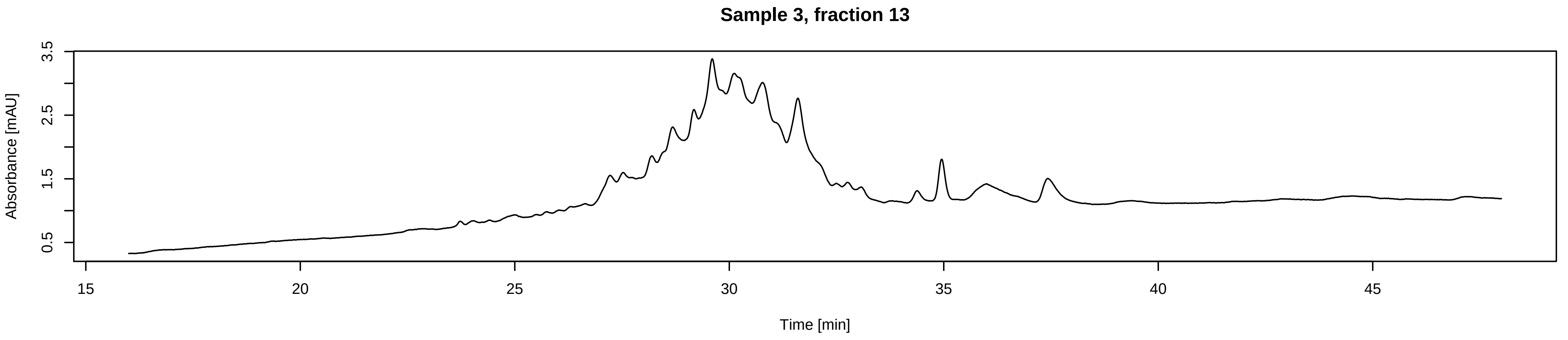
Sample 3, fraction 13
Absorbance [mAU]
0.5
1.5
2.5
3.5
15
20
25
30
35
40
45
Time [min]

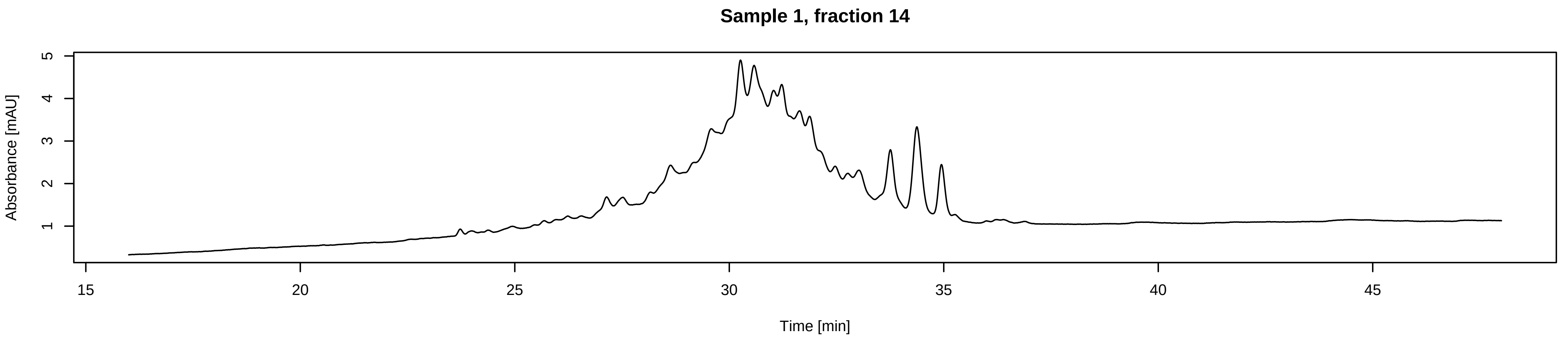
Sample 1, fraction 14
Absorbance [mAU]
1
2
3
4
5
15
20
25
30
35
40
45
Time [min]

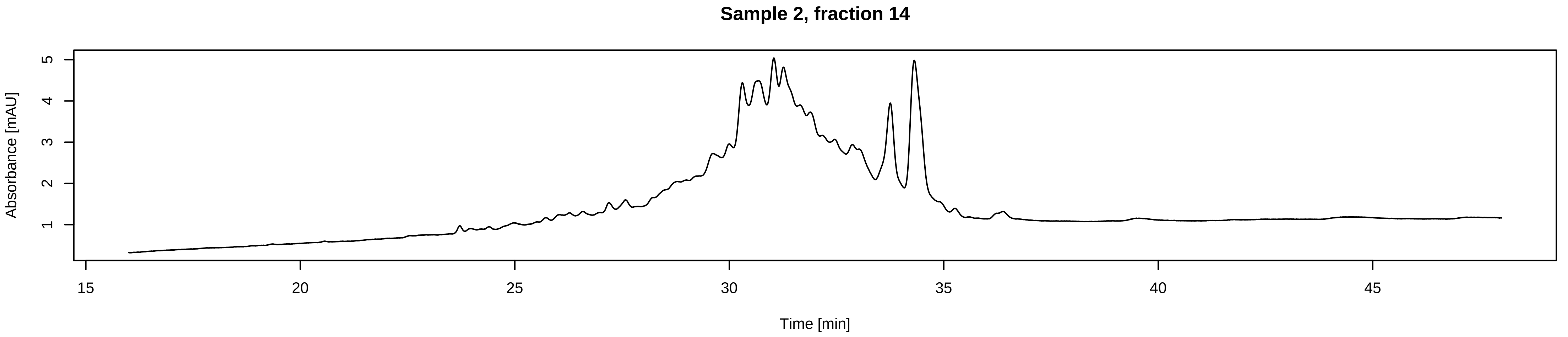
Sample 2, fraction 14
Absorbance [mAU]
1
2
3
4
5
15
20
25
30
35
40
45
Time [min]

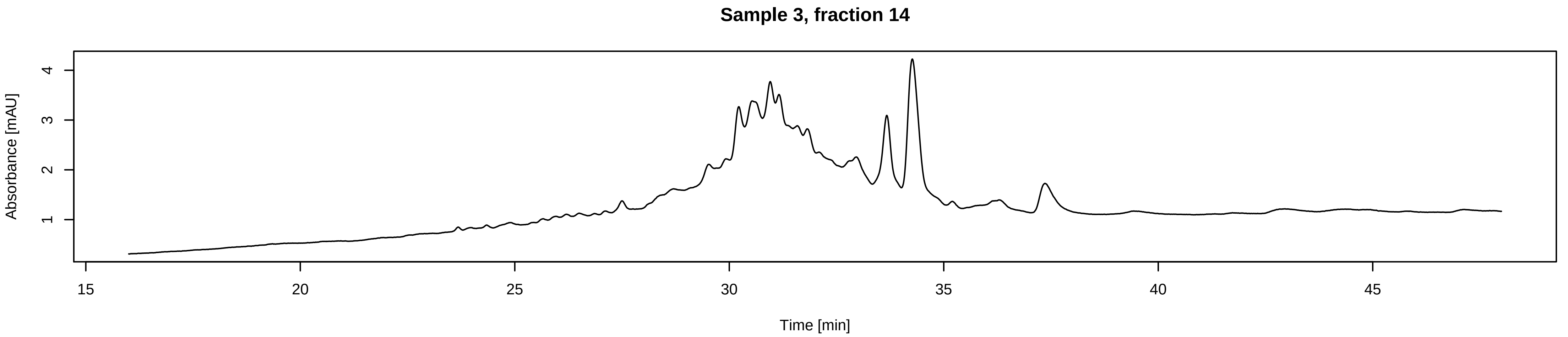
Sample 3, fraction 14
Absorbance [mAU]
1
2
3
4
15
20
25
30
35
40
45
Time [min]

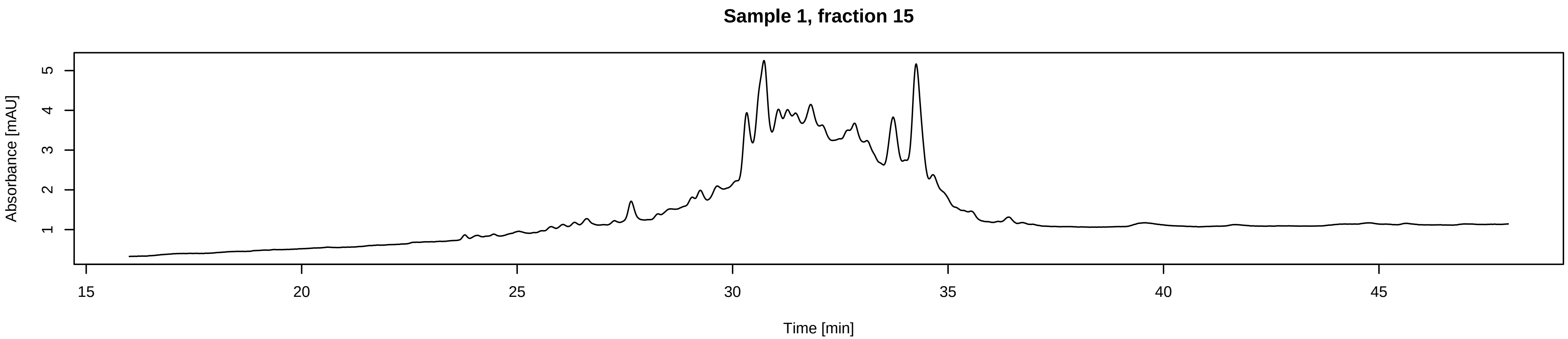
Sample 1, fraction 15
Absorbance [mAU]
1
2
3
4
5
15
20
25
30
35
40
45
Time [min]

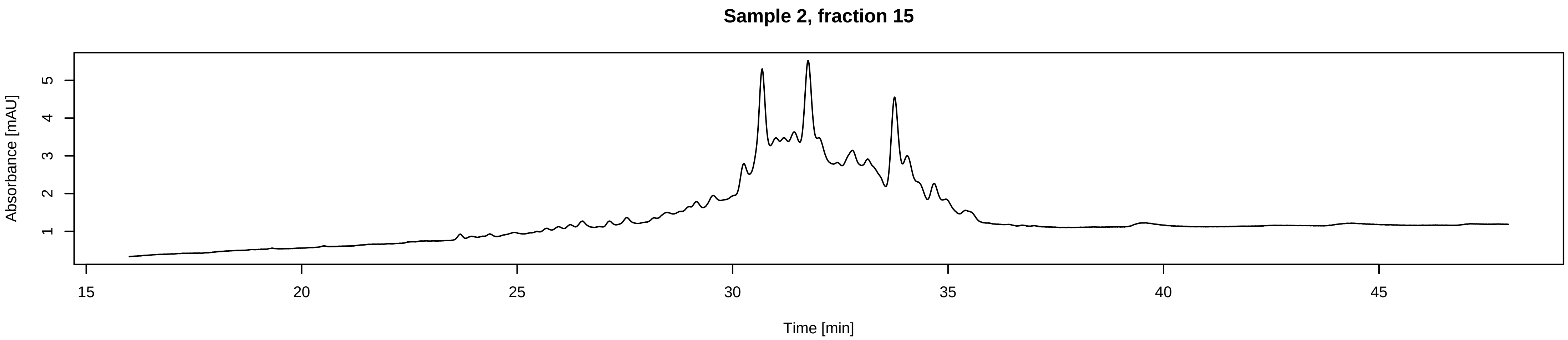
Sample 2, fraction 15
Absorbance [mAU]
1
2
3
4
5
15
20
25
30
35
40
45
Time [min]

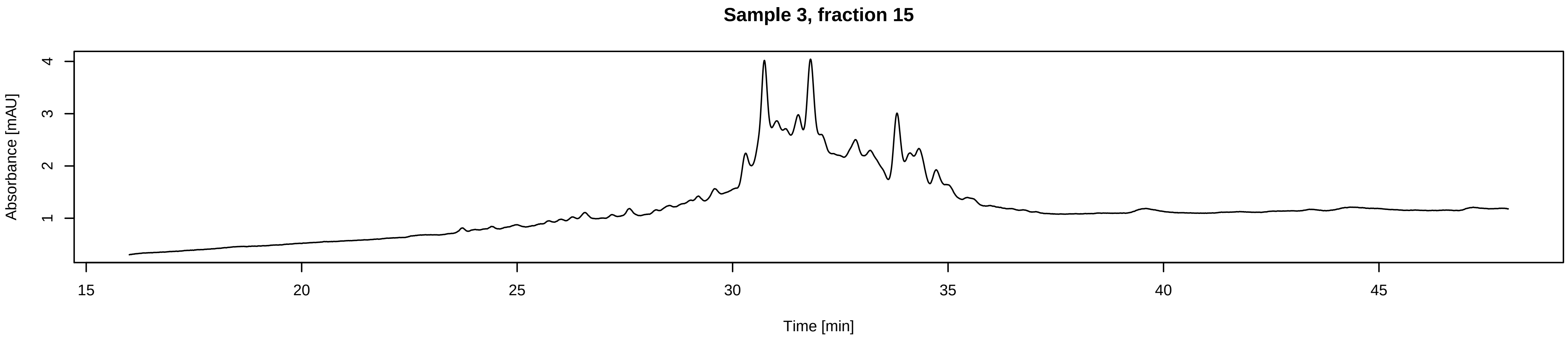
Sample 3, fraction 15
Absorbance [mAU]
1
2
3
4
15
20
25
30
35
40
45
Time [min]

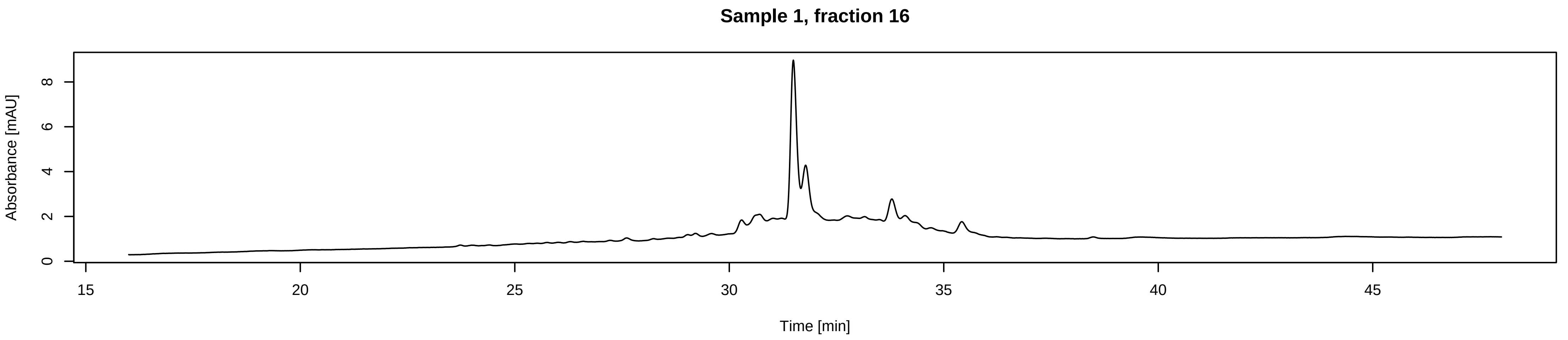
Sample 1, fraction 16
Absorbance [mAU]
0
2
4
6
8
15
20
25
30
35
40
45
Time [min]

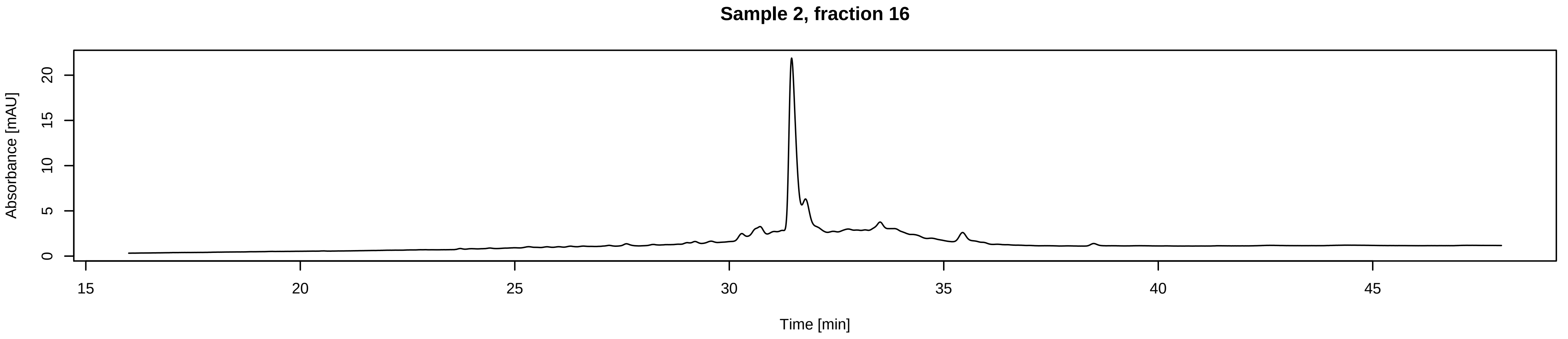
Sample 2, fraction 16
Absorbance [mAU]
0
5
10
15
20
15
20
25
30
35
40
45
Time [min]

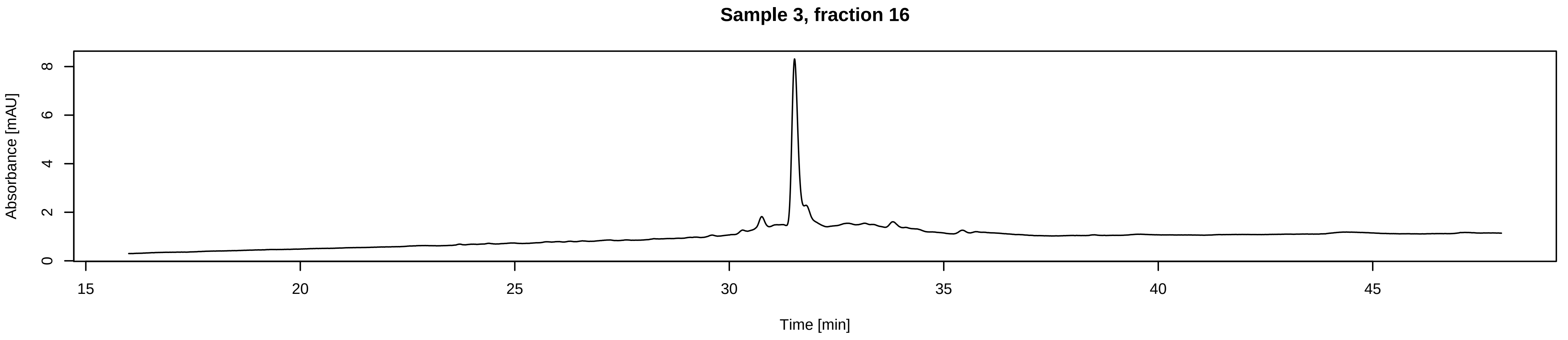
Sample 3, fraction 16
Absorbance [mAU]
0
2
4
6
8
15
20
25
30
35
40
45
Time [min]

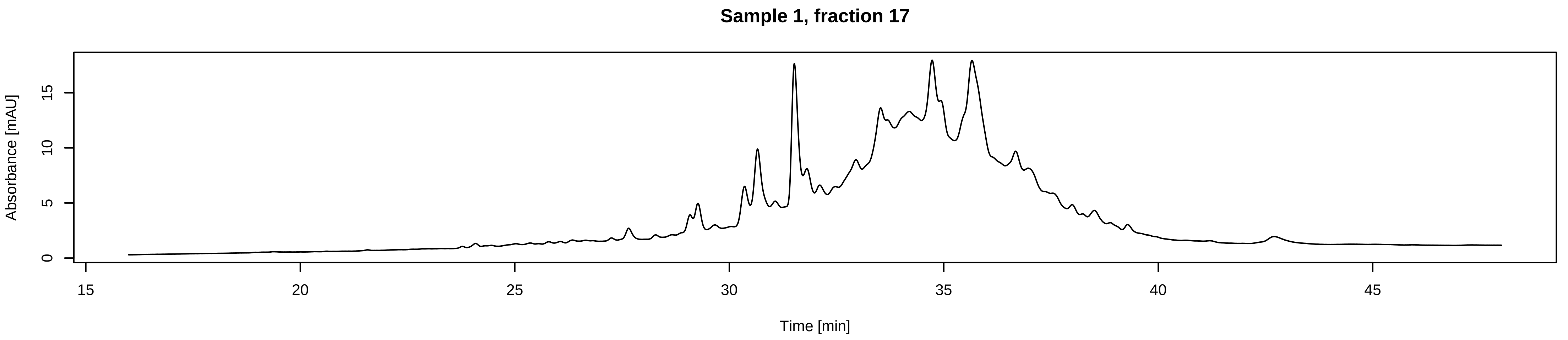
Sample 1, fraction 17
Absorbance [mAU]
0
5
10
15
15
20
25
30
35
40
45
Time [min]

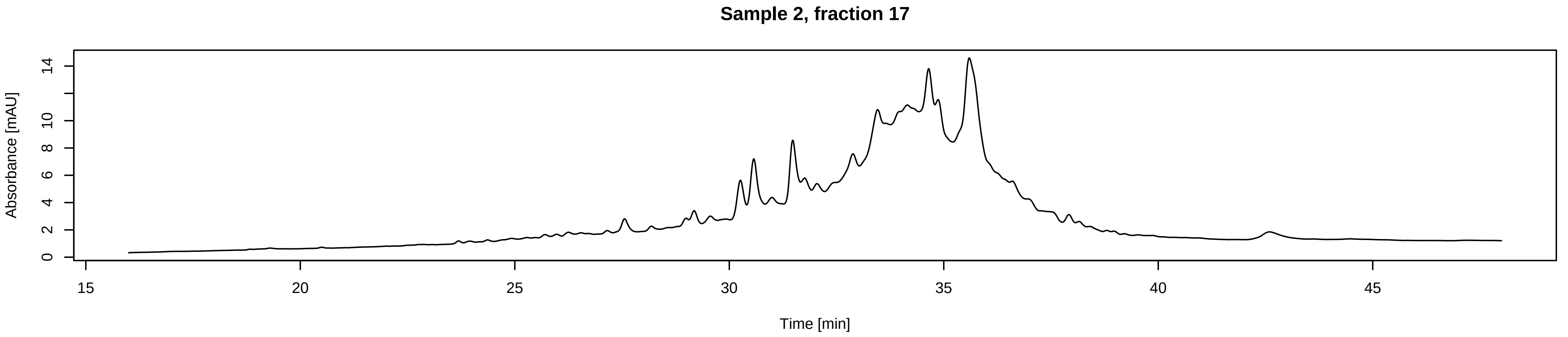
Sample 2, fraction 17
Absorbance [mAU]
0
2
4
6
8
10
14
15
20
25
30
35
40
45
Time [min]

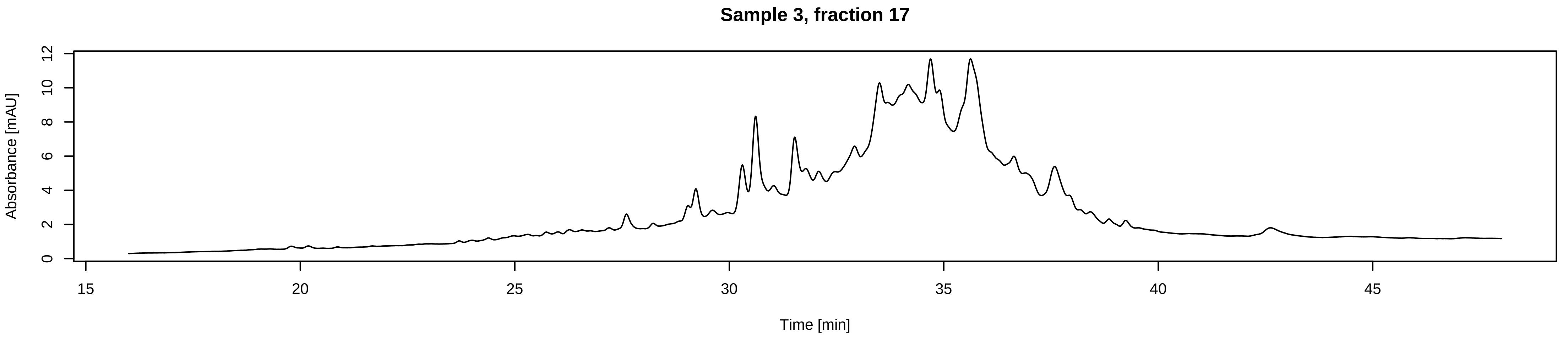
Sample 3, fraction 17
Absorbance [mAU]
0
2
4
6
8
10
12
15
20
25
30
35
40
45
Time [min]

Table S1. Summary statistics of phosphopeptides identified in each replicate of purified and fractionated sample

		Exclusive	Total
Replicate 1	Number of peptides in purified sample	556	4554
	Number of peptides in fractionated sample	3121	10128
	**Fold change**		**2.22**
	Number of phosphopeptides in purified sample	593	2286
	Number of phosphopeptides in fractionated sample	3602	5295
	**Fold change**		**2.32**
	Number of shared phosphopeptides	1693	
	Number of shared peptides in total	3405	
Replicate 2	Number of peptides in purified sample	496	4130
	Number of peptides in fractionated sample	4244	12647
	**Fold change**		**3.06**
	Number of phosphopeptides in purified sample	259	1904
	Number of phosphopeptides in fractionated sample	5028	6673
	**Fold change**		**3.50**
	Number of shared phosphopeptides	1645	
	Number of shared peptides in total	3375	
Replicate 3	Number of peptides in purified sample	524	3873
	Number of peptides in fractionated sample	2891	10653
	**Fold change**		**2.75**
	Number of phosphopeptides in purified sample	280	1764
	Number of phosphopeptides in fractionated sample	4693	6177
	**Fold change**		**3.50**
	Number of shared phosphopeptides	1484	
	Number of shared peptides in total	3069	
	CV for peptides in the purified sample	8.22%	
	CV for phosphopeptides in the purified sample	13.61%	
	CV for peptides in the fractionated sample	11.93%	
	CV for phosphopeptides in the fractionated sample	11.54%	

CV stands for coefficient of variation
